# Secondhand smoke exposure among non-smoking adolescents in three Vietnamese cities in 2025: A cross-sectional study

**DOI:** 10.18332/tid/224237

**Published:** 2026-07-31

**Authors:** Hoang Le Tu, Thao Nguyen Thi Bich, Quynh Anh Nguyen, Tan Luong Minh, Trang Phan Thi Thu, Quynh Chi Nguyen Thai, Nga Nguyen Thi, Mai Tran Thi Hoa, Hoang Minh Dao Le, Lam Nguyen Tuan, Meheret Melles-Brewer, Huong Le Thi Thanh

**Affiliations:** 1 Hanoi University of Public Health, Hanoi, Vietnam; 2 Graduate School of Cancer Science and Policy, National Cancer Center, Goyang, South Korea; 3 World Health Organization, Vietnam Office, Hanoi, Vietnam

**Keywords:** secondhand smoke, adolescents, tobacco, cross-sectional study, Vietnam

## Abstract

**INTRODUCTION:**

Secondhand smoke (SHS) remains common among adolescents, but evidence on setting-specific exposure in Vietnam is limited. This study estimated the prevalence of any and setting-specific SHS exposure from conventional cigarettes among non-current smoking students in three Vietnamese cities and identified associated factors.

**METHODS:**

We conducted a secondary analysis of self-reported questionnaire data from a school-based cross-sectional survey in Hanoi, Da Nang, and Ho Chi Minh City in 2025. The analysis included non-current smoking students aged 13 to under 18 years. The primary outcome was any SHS exposure in the past 7 days, defined as exposure at home, in indoor public places, or in outdoor public places. Secondary outcomes were setting-specific exposures. Associations were examined using Poisson regression with robust standard errors clustered at the school level, reported as adjusted prevalence ratios (APRs) and 95% confidence intervals (CIs).

**RESULTS:**

Among 4437 non-current smoking students, 67.2% reported any SHS exposure, 22.0% reported home exposure, 50.4% reported indoor public-place exposure, and 57.5% reported outdoor public-place exposure. Indoor smoking at home was the strongest factor associated with any SHS exposure (APR=1.62; 95% CI: 1.55–1.70). Higher prevalence was also observed among male students (APR=1.05; 95% CI:1.01–1.10), Grade 12 students (APR=1.15; 95% CI:1.06–1.25), students in Ho Chi Minh City (APR=1.08; 95% CI: 1.01–1.15), and selected weekly allowance groups, which may reflect greater mobility and spending autonomy. Setting-specific analyses showed lower home exposure but higher public-place exposure among older students.

**CONCLUSIONS:**

SHS exposure was highly prevalent among non-current smoking students in three major Vietnamese cities and varied by household and public-place contexts. Further longitudinal and implementation studies are needed to clarify causal pathways and strengthen the evidence base for strategies to reduce adolescent SHS exposure in home and public environments.

## INTRODUCTION

Secondhand smoke (SHS) remains a major public health problem particularly for children and adolescents who are exposed involuntarily and are biologically more vulnerable to its harmful effects^1-3^. Recent global evidence shows that SHS continues to contribute substantially to disease burden among non-smokers, especially in low- and middle-income countries, and there is no safe level of exposure^1,4^. In children and adolescents, SHS exposure has been linked to adverse respiratory outcomes, asthma exacerbation, respiratory infections, and other short- and long-term health harms^1-3^. Despite decades of tobacco control efforts, SHS exposure among young people remains common worldwide^5,6^.

Recognizing these risks, the World Health Organization Framework Convention on Tobacco Control (WHO FCTC) recommends comprehensive measures to protect people from exposure to tobacco smoke, including implementing 100% smoke-free indoor public places, workplaces, and public transport under Article 8^7^. The WHO MPOWER package further emphasizes protecting people from SHS as a core tobacco control strategy^8^. Vietnam signed the WHO FCTC in August 2003, ratified it in November 2004, and subsequently enacted the Law on Prevention and Control of Tobacco Harms in 2012, which introduced smoke-free requirements in healthcare, educational, and indoor public venues^9^.

Recent international studies indicate that adolescent SHS exposure is still widespread across countries and settings. A pooled analysis of 122 countries using Global Youth Tobacco Survey data reported that SHS exposure affected a large proportion of adolescents globally, with substantial exposure both at home and in public places^5^. Other analyses of repeated cross-sectional surveys have shown that, although SHS exposure at home has declined in some settings, exposure in public places has often remained stable or decreased more slowly, suggesting incomplete protection of adolescents from tobacco smoke in everyday environments^6,10^. Evidence from the United States further suggests that measuring indoor and outdoor public exposure separately provides important information, because both settings contribute meaningfully to overall youth exposure^11^.

The household smoking status remains especially important. Research over the last few years has consistently shown that smoking inside the home is a major predictor of SHS exposure among children and adolescents^2,3,12^. This is particularly concerning because smoke-free legislation in public places does not protect children from exposure in private settings. At the same time, adolescent SHS exposure is not confined to the household. Public-place exposure remains common, especially in urban and socially active populations, indicating that both household and community environments shape exposure risk^6,11^.

In Vietnam, tobacco control policies have been implemented for more than a decade, including smoke-free regulations in a range of public places^9^. Supporting regulations and implementation guidelines have further strengthened smoke-free policies; however, enforcement and compliance remain variable, particularly in environments frequently accessed by adolescents^13-16^. A Vietnamese study assessing public venues, five years after implementation of the tobacco control law, found that SHS concentrations had declined but exposure in public places remained detectable and enforcement gaps persisted^13^. More recent survey-based analyses in Vietnam have shown that SHS exposure remains common among adults and varies by sociodemographic factors. In the 2022 Vietnam Provincial Global Adult Tobacco Survey, 44.4% of adults were exposed to SHS at home and 23.1% at work; exposure was higher among women at home, among younger adults aged 15–24 years, and among urban residents (OR=1.15; 95% CI: 1.08–1.22)^17^. However, adolescent-specific evidence in Vietnam remains limited, especially evidence that distinguishes exposure at home, in indoor public places, and in outdoor public places.

This gap is important because adolescents may experience SHS differently depending on age, mobility, family environment, and urban context. Older students may spend more time outside the home and therefore encounter more exposure in public settings, whereas younger students may be more affected by household smoking. In addition, factors such as household smoking, city of residence, and spending autonomy may shape exposure in different ways across settings^5,6,10-12^. Understanding these patterns is important for designing tobacco control interventions that are relevant to adolescents in large cities. Therefore, this study aimed to estimate the prevalence of any and setting-specific SHS exposure from conventional cigarettes among non-current smoking students aged 13 to under 18 years in three selected cities in Vietnam, and to identify factors associated with these exposures.

## METHODS

### Study design and setting

This study was a secondary analysis of the quantitative component of a larger mixed-methods school-based survey conducted in Hanoi, Da Nang, and Ho Chi Minh City, Vietnam, from mid-October to the first week of December 2025. The parent study included quantitative, qualitative, and observational components; the present study used the cross-sectional student survey only. The three cities were selected to represent the northern, central, and southern regions of Vietnam.

### Sample size and sampling

The target population for the quantitative survey was students aged 13–17 years currently enrolled in secondary and high schools in the three study cities. The required sample size was calculated using a formula for estimating a single population proportion, assuming a prevalence of 8% for current e-cigarette use among students in 2020^18^, a 95% confidence level, a design effect of 2, and absolute precision of 0.02. This yielded a minimum sample of 1414 students per city; after adding 10% for invalid questionnaires or non-response, the target sample was 4665 students across the three cities. After excluding invalid or incomplete questionnaires, 4475 valid questionnaires were available for analysis in the parent survey.

A two-stage cluster sampling design was used. In the first stage, schools within each city were selected by probability proportional to size (PPS) based on eligible enrollment. Secondary schools contributed students from Grades 8 and 9, and high schools from Grades 10–12. In the second stage, classes were selected by simple random sampling within each selected school. Two classes were selected in each secondary school, and three classes in each high school, and all students in the selected classes were invited to participate.

For the present analysis, we further restricted the sample to non-current smoking students, defined as students classified in the dataset as never smokers or former smokers, and excluding current cigarette smokers.

### Data collection

After ethics approval and administrative permission were obtained, trained investigators introduced the study to students in class and explained the questionnaire and completion procedures. Students then self-completed the questionnaire. The questionnaire was developed based on the Global Youth Tobacco Survey 2023 and the Vietnam Youth Tobacco Survey and included domains on sociodemographic characteristics, knowledge and attitudes, conventional cigarette use and secondhand smoke exposure, e-cigarette use, heated tobacco product use, advertising and promotion, cessation, and mental health. The questionnaire was piloted in one secondary school class and one high school class in Hanoi; the two pilot schools were not included in the final survey.

### Variables and measurements

*Outcome variables*

The primary outcome for this analysis was any secondhand smoke (SHS) exposure from conventional cigarettes in the past 7 days. This variable was derived from three questionnaire items asking whether, during the past 7 days, someone had smoked conventional cigarettes inside the student’s home, in indoor public places, or in outdoor public places when the student was present. Each source variable was recoded into a binary exposure measure, with zero days coded as no exposure and ≥1 day coded as exposure. A composite binary variable, any SHS exposure, was then created and defined as exposure in at least one of these three settings.

Secondary outcomes were three setting-specific binary variables. Home SHS exposure was defined by the item assessing cigarette smoking inside the student’s home in the past 7 days. Indoor public-place SHS exposure was defined from the item assessing cigarette smoking in indoor public places during the same period. Outdoor public-place SHS exposure was defined from the corresponding item on outdoor public places. Each of these variables was dichotomized using the same rule as the primary outcome, with zero days indicating no exposure and ≥1 day indicating exposure.

*Explanatory variables*

Explanatory variables were selected *a priori* based on conceptual relevance to adolescent SHS exposure, previous evidence, and the variables available in the questionnaire and codebook^5,6,10-12^. Sex, city, grade, living arrangement, and weekly allowance were considered potential confounding factors because they may be associated with both adolescents’ likelihood of SHS exposure and their household or public-environment contexts^5,6,10,11^. Indoor smoking at home was included as a key household smoking-environment variable and was also considered a potential confounding factor in models of public-place SHS exposure^12^.

Sex was categorized as female or male. City was categorized as Hanoi, Da Nang, or Ho Chi Minh City. Grade was categorized from Grade 8 to Grade 12. Living arrangement was categorized as living with both parents, living with only the father or mother, living with relatives, or living in a dormitory or boarding arrangement. Weekly allowance (in Vietnamese Dong, VND) was categorized as none, <50000, 50000 to <100000, 100000 to <200000, 200000 to <500000, or ≥500000.

Indoor smoking at home was derived from a questionnaire item assessing smoking inside the home and was coded as a binary variable. Because this variable overlapped conceptually with the secondary outcome of home SHS exposure, it was not included in the multivariable model for the home-specific outcome. However, it was retained in the models for any place SHS exposure, indoor public-place SHS exposure, and outdoor public-place SHS exposure.

### Statistical analysis

Descriptive analyses were used to summarize participant characteristics and the prevalence of any and setting-specific SHS exposure. Categorical variables were presented as numbers and percentages. Bivariate comparisons of student characteristics by any SHS exposure status were assessed using chi-squared tests.

Because the outcomes were binary and common, Poisson regression with a log link was preferred over logistic regression to estimate prevalence ratios directly. Logistic regression produces odds ratios, which can overestimate prevalence ratios when outcomes are common, whereas modified Poisson regression with robust standard errors is an accepted approach for estimating adjusted prevalence ratios in cross-sectional studies^19,20^. Robust standard errors clustered at the school level were used to account for within-school association arising from the cluster sampling design. Results were reported as adjusted prevalence ratios (APRs) with 95% confidence intervals (CIs). For the primary outcome of any place SHS exposure, two multivariable models were fitted. Model 1 adjusted for demographic and social factors, including sex, city, grade, living arrangement, and weekly allowance. Model 2 additionally adjusted for indoor smoking at home.

For the secondary analyses, separate multivariable models were fitted for home SHS exposure, indoor public-place SHS exposure, and outdoor public-place SHS exposure. Indoor smoking at home was excluded from the home-specific model because of conceptual overlap with the outcome. All analyses were conducted in Stata BE 19.5.

Because the analytic dataset did not include sampling weights, weighted population estimates could not be calculated; therefore, descriptive estimates are presented as unweighted sample estimates. Multivariable analyses were conducted using complete-case analysis, and the analytic sample size varied slightly across models because of missing covariate data. Robust standard errors clustered at the school level were used to account for within-school correlation arising from the cluster sampling design.

### Ethical considerations

This study received ethical approval from the Institutional Review Board of the Hanoi University of Public Health under Decision No. 475/2025/YTCC-HD3, dated 10 October 2025. Participation was voluntary, and consent procedures involved both students and their parents or legal guardians. Permissions were also obtained from participating schools and relevant education authorities before data collection.

## RESULTS

### Characteristics of the study participants

Table 1 summarizes the characteristics of 4437 non-current smoking students included in the analysis. The sample was nearly balanced by sex, with 49.1% female and 50.9% male. Students were also relatively evenly distributed across the three study cities, with 32.6% from Hanoi, 33.4% from Da Nang, and 34.0% from Ho Chi Minh City. Grade 8 students comprised the largest proportion of the sample (22.6%), while the remaining grades each accounted for approximately one-fifth of participants. Most students lived with both parents (84.9%). More than one-third reported having no weekly allowance (34.7%), whereas 20.6% reported receiving <50000 VND per week. Nearly half of the students (46.1%) reported indoor smoking at home.

**Table 1:** Characteristics of non-current smoking students in Hanoi, Da Nang, and Ho Chi Minh City, Vietnam, 2025 (N=4437)

*Characteristics*	*n (%)*
**Sex**	
Female	2171 (49.1)
Male	2254 (50.9)
**City**	
Hanoi	1447 (32.6)
Da Nang	1480 (33.4)
Ho Chi Minh City	1510 (34.0)
**Grade**	
8	1001 (22.6)
9	826 (18.6)
10	877 (19.8)
11	879 (19.8)
12	854 (19.2)
**Living arrangement**	
Both parents	3763 (84.9)
Father/mother	423 (9.5)
Relatives	158 (3.6)
Dormitory/boarding	88 (2.0)
**Weekly allowance** (VND)	
None	1537 (34.7)
<50000	914 (20.6)
50000 to <100000	803 (18.1)
100000 to <200000	625 (14.1)
200000 to <500000	409 (9.2)
≥500000	143 (3.2)
**Indoor smoking at home**	
No	2388 (53.9)
Yes	2040 (46.1)

Percentages are based on non-missing responses for each variable; therefore, totals may vary. VND: 1 million Vietnamese Dong about US$38.

### Prevalence of secondhand smoke exposure among study participants

Table 2 shows the prevalence of any and setting-specific SHS exposure from conventional cigarettes among non-current smoking students. Overall, 67.2% of students reported any SHS exposure in the past 7 days. Outdoor public-place exposure was the most common setting-specific exposure (57.5%), followed by indoor public-place exposure (50.4%), whereas home exposure was reported by 22.0% of students. The prevalence of any SHS exposure was higher among students in Da Nang (69.7%) and Ho Chi Minh City (69.8%) than in Hanoi (61.4%), and increased across grade levels, reaching 72.4% among Grade 12 students. Students with higher weekly allowance generally had higher prevalence of SHS exposure than those reporting no allowance, ranging from 68.8% to 74.3% across allowance categories compared with 59.7% among students with no allowance. The largest contrast was observed by household smoking environment: students reporting indoor smoking at home had a higher prevalence of any SHS exposure than those without indoor smoking at home (84.9% vs 52.1%).

**Table 2:** Prevalence of any and setting-specific secondhand smoke exposure from conventional cigarettes among non-current smoking students in Hanoi, Da Nang, and Ho Chi Minh City, Vietnam, 2025 (N=4437)

*Variables*	*Place of SHS exposure*			
	*Any place* *n (%)*	*At home* *n (%)*	*Indoor public place* *n (%)*	*Outdoor public place* *n (%)*
**Total**	2975 (67.2)	975 (22.0)	2233 (50.4)	2548 (57.5)
**Sex**				
Female	1415 (65.2)	467 (21.5)	1079 (49.7)	1216 (56.0)
Male	1551 (68.8)	504 (22.4)	1146 (50.8)	1326 (58.8)
**City**				
Hanoi	889 (61.4)	286 (19.8)	673 (46.5)	735 (50.8)
Da Nang	1032 (69.7)	386 (26.1)	799 (54.0)	867 (58.6)
Ho Chi Minh City	1054 (69.8)	303 (20.1)	761 (50.4)	946 (62.6)
**Grade**				
8	619 (61.8)	243 (24.3)	430 (43.0)	504 (50.3)
9	562 (68.0)	215 (26.0)	420 (50.8)	480 (58.1)
10	571 (65.1)	177 (20.2)	432 (49.3)	476 (54.3)
11	605 (68.8)	184 (20.9)	457 (52.0)	525 (59.7)
12	618 (72.4)	156 (18.3)	494 (57.8)	563 (65.9)
**Living arrangement**				
Both parents	2541 (67.5)	830 (22.1)	1896 (50.4)	2179 (57.9)
Father/mother	289 (68.3)	93 (22.0)	221 (52.2)	251 (59.3)
Relatives	89 (56.3)	38 (24.1)	72 (45.6)	72 (45.6)
Dormitory/boarding	54 (61.4)	13 (14.8)	43 (48.9)	46 (52.3)
**Weekly allowance** (VND)				
None	917 (59.7)	297 (19.3)	685 (44.6)	749 (48.7)
<50000	629 (68.8)	221 (24.2)	454 (49.7)	529 (57.9)
50000 to <100000	573 (71.4)	170 (21.2)	421 (52.4)	506 (63.0)
100000 to <200000	449 (71.8)	149 (23.8)	341 (54.6)	396 (63.4)
200000 to <500000	304 (74.3)	103 (25.2)	248 (60.6)	276 (67.5)
≥500000	99 (69.2)	32 (22.4)	82 (57.3)	90 (62.9)
**Indoor smoking at home**				
No	1243 (52.1)	110 (4.6)	850 (35.6)	1115 (46.7)
Yes	1732 (84.9)	865 (42.4)	1383 (67.8)	1433 (70.2)

SHS: secondhand smoke. VND: 1 million Vietnamese Dong about US$38.

### Factors associated with any SHS exposure and setting-specific SHS exposure

Table 3 compares the characteristics of students according to whether they reported any SHS exposure from conventional cigarettes. Compared with unexposed students, those with SHS exposure were more often male (52.3% vs 48.1%, p=0.008), more likely to live in Da Nang (34.7% vs 30.7%) or Ho Chi Minh City (35.4% vs 31.1%) rather than Hanoi (29.9% vs 38.2%) with p<0.001, and more likely to be in higher grades, particularly Grade 11 (20.3% vs 18.7%) and Grade 12 (20.8% vs 16.2%) (p<0.001). Differences were also observed by living arrangement and weekly allowance, with exposed students less likely to report no allowance (30.9% vs 42.4%) and more likely to report higher weekly allowance levels (p<0.001). The greatest disparity was seen in household smoking environment: indoor smoking at home was reported by 58.2% of students with SHS exposure, compared with 21.2% of those without SHS exposure (p<0.001).

**Table 3:** Characteristics of non-current smoking students by any secondhand smoke exposure from conventional cigarettes in Hanoi, Da Nang, and Ho Chi Minh City, Vietnam, 2025

*Variables*	*Any SHS exposure to conventional cigarettes*		
	*No (N=1455)*	*Yes (N=2975)*	p*
**Total**	1455 (32.8)	2975 (67.2)	
**Sex**			0.008
Female	754 (51.9)	1415 (47.7)	
Male	698 (48.1)	1551 (52.3)	
**City**			<0.001
Hanoi	556 (38.2)	889 (29.9)	
Da Nang	447 (30.7)	1032 (34.7)	
Ho Chi Minh City	452 (31.1)	1054 (35.4)	
**Grade**			<0.001
8	379 (26.0)	619 (20.8)	
9	263 (18.1)	562 (18.9)	
10	306 (21.0)	571 (19.2)	
11	272 (18.7)	605 (20.3)	
12	235 (16.2)	618 (20.8)	
**Living arrangement**			0.020
Both parents	1217 (83.8)	2541 (85.5)	
Father/mother	133 (9.2)	289 (9.7)	
Relatives	68 (4.7)	89 (3.0)	
Dormitory/boarding	34 (2.3)	54 (1.8)	
**Weekly allowance** (VND)			<0.001
None	617 (42.4)	917 (30.9)	
<50000	285 (19.6)	629 (21.2)	
50000 to <100000	229 (15.7)	573 (19.3)	
100000 to <200000	175 (12.0)	449 (15.1)	
200000 to <500000	105 (7.2)	304 (10.2)	
≥500000	43 (3.0)	99 (3.3)	
**Indoor smoking at home**			<0.001
No	1,145 (78.8)	1243 (41.8)	
Yes	308 (21.2)	1732 (58.2)	

Data are presented as n (% column).

* Derived from chi-squared tests. SHS: secondhand smoke. VND: 1 million Vietnamese Dong about US$38.

Table 4 shows that, after adjustment, indoor smoking at home was the strongest factor associated with any SHS exposure from conventional cigarettes. In Model 2, students reporting indoor smoking at home had a 62% higher prevalence of any SHS exposure than those without indoor smoking at home (APR=1.62; 95% CI: 1.55–1.70). Male students also had a slightly higher prevalence than female students (APR=1.05; 95% CI: 1.01–1.10), and students in Ho Chi Minh City had a higher prevalence than those in Hanoi (APR=1.08; 95% CI: 1.01–1.15). Compared with Grade 8 students, Grade 12 students were more likely to report SHS exposure (APR=1.15; 95% CI: 1.06–1.25). Weekly allowance (VND) was also positively associated with SHS exposure, particularly among students receiving 50000 to <100000 (APR=1.17; 95% CI: 1.12–1.22) and 200000 to <500000 (APR=1.15; 95% CI: 1.06–1.24). In contrast, living with relatives was associated with lower prevalence of SHS exposure than living with both parents (APR=0.84; 95% CI: 0.75–0.95).

**Table 4:** Factors associated with any secondhand smoke exposure from conventional cigarettes among non-current smoking students in Hanoi, Da Nang, and Ho Chi Minh City, Vietnam, 2025

*Variables*	*Model 1*			*Model 2*		
	*APR*	*95% CI*	*p*	*APR*	*95% CI*	*p*
**Sex**						
Female (ref.)						
Male	1.05*	1.01–1.09	0.023	1.05*	1.01–1.10	0.017
**City**						
Hanoi (ref.)						
Da Nang	1.10**	1.03–1.17	0.002	1.05	0.99–1.11	0.100
Ho Chi Minh City	1.08*	1.01–1.15	0.022	1.08*	1.01–1.15	0.027
**Grade**						
8 (ref.)						
9	1.10	1.00–1.21	0.062	1.09	1.00–1.20	0.063
10	1.03	0.94–1.13	0.545	1.04	0.95–1.13	0.414
11	1.09	1.00–1.18	0.056	1.08	0.99–1.18	0.092
12	1.13**	1.04–1.24	0.007	1.15**	1.06–1.25	0.001
**Living arrangement**						
Both parents (ref.)						
Father/mother	1.00	0.92–1.07	0.900	1.01	0.95–1.09	0.684
Relatives	0.85*	0.73–0.97	0.019	0.84**	0.75–0.95	0.005
Dormitory/ boarding	0.92	0.80–1.06	0.273	0.96	0.85–1.08	0.491
**Weekly allowance** (VND)						
None (ref.)						
<50000	1.13***	1.06–1.21	<0.001	1.11***	1.05–1.17	<0.001
50000 to <100000	1.16***	1.10–1.22	<0.001	1.17***	1.12–1.22	<0.001
100000 to <200000	1.17***	1.10–1.24	<0.001	1.14***	1.08–1.21	<0.001
200000 to <500000	1.19***	1.09–1.30	<0.001	1.15***	1.06–1.24	0.001
≥500000	1.13	0.98–1.30	0.090	1.11	0.97–1.26	0.123
**Indoor smoking at home**						
No (ref.)						
Yes				1.62***	1.55–1.70	<0.001
Observations	4412			4410		

Model 1: adjusted for demographic and social factors. Model 2 was additionally adjusted for indoor smoking at home. The total analytic sample was 4437 non-current smoking students. Model-specific observations are based on complete-case analysis and may vary because of missing covariate data. Poisson regression with log link and standard errors clustered at the school level. APR: adjusted prevalence ratio. SHS: secondhand smoke. VND: 1 million Vietnamese Dong about US$38.

* p<0.05,

** p<0.01,

*** p<0.001.

Table 5 shows that factors associated with SHS exposure by setting. For home SHS exposure, prevalence was higher among students in Da Nang than in Hanoi (APR=1.29; 95% CI: 1.07–1.56) and lower among students in Grades 10–12 than among Grade 8 students, with the lowest prevalence observed in Grade 12 (APR=0.70; 95% CI: 0.56–0.87). Higher weekly allowance (VND) was also associated with home SHS exposure, particularly among students receiving 200000 to <500000 (APR=1.49; 95% CI: 1.20–1.86). For public-place exposure, Grade 12 students had higher prevalence of both indoor public-place SHS exposure (APR=1.31; 95% CI: 1.15–1.49) and outdoor public-place SHS exposure (APR=1.26; 95% CI: 1.13–1.41) than Grade 8 students. Outdoor public-place SHS exposure was also higher in Ho Chi Minh City than in Hanoi (APR=1.14; 95% CI: 1.05–1.25), while living with relatives was associated with lower outdoor public-place SHS exposure than living with both parents (APR=0.80; 95% CI: 0.69–0.91). Indoor smoking at home was associated with both indoor public-place SHS exposure (APR=1.89; 95% CI: 1.77–2.02) and outdoor public-place SHS exposure (APR=1.50; 95% CI: 1.42–1.58).

**Table 5:** Factors associated with setting-specific secondhand smoke exposure from conventional cigarettes among non-current smoking students in Hanoi, Da Nang, and Ho Chi Minh City, Vietnam, 2025

*Variables*	*Home*			*Indoor public place*			*Outdoor public place*		
	*APR*	*95% CI*	*p*	*APR*	*95% CI*	*p*	*APR*	*95% CI*	*p*
**Sex**									
Female (ref.)									
Male	1.02	0.92–1.14	0.665	1.03	0.97–1.09	0.389	1.04	0.99–1.10	0.132
**City**									
Hanoi (ref.)									
Da Nang	1.29**	1.07–1.56	0.007	1.06	0.97–1.15	0.202	1.06	0.97–1.16	0.178
Ho Chi Minh City	0.94	0.77–1.15	0.559	1.01	0.93–1.10	0.789	1.14**	1.05–1.25	0.003
**Grade**									
8 (ref.)									
9	1.04	0.91–1.19	0.540	1.17*	1.02–1.33	0.025	1.15*	1.02–1.29	0.025
10	0.78*	0.65–0.95	0.012	1.12	0.99–1.26	0.061	1.05	0.94–1.18	0.408
11	0.81*	0.66–0.99	0.040	1.15*	1.01–1.31	0.033	1.14*	1.00–1.29	0.042
12	0.70**	0.56–0.87	0.002	1.31***	1.15–1.49	0.000	1.26***	1.13–1.41	<0.001
**Living arrangement**									
Both parents (ref.)									
Father/mother	0.97	0.80–1.18	0.758	1.04	0.96–1.13	0.301	1.02	0.93–1.12	0.689
Relatives	1.09	0.84–1.41	0.526	0.90	0.77–1.06	0.224	0.80**	0.69–0.91	0.001
Dormitory/boarding	0.73	0.46–1.17	0.190	1.03	0.85–1.25	0.752	0.97	0.83–1.12	0.648
**Weekly allowance** (VND)									
None (ref.)									
<50000	1.22*	1.01–1.47	0.037	1.08	0.98–1.18	0.107	1.14**	1.05–1.24	0.003
50000 to <100000	1.13	0.96–1.33	0.145	1.16***	1.07–1.25	<0.001	1.24***	1.15–1.34	<0.001
100000 to <200000	1.33**	1.12–1.59	0.001	1.17***	1.09–1.25	<0.001	1.21***	1.13–1.30	<0.001
200000 to <500000	1.49***	1.20–1.86	<0.001	1.25***	1.14–1.37	<0.001	1.23***	1.13–1.35	<0.001
≥500000	1.29	0.88–1.88	0.187	1.23**	1.07–1.41	0.003	1.20*	1.04–1.39	0.014
**Indoor smoking at home**									
No (ref.)									
Yes				1.89***	1.77–2.02	<0.001	1.50***	1.42–1.58	<0.001
Observations	4413			4408			4409		

The total analytic sample was 4437 non-current smoking students. Model-specific observations are based on complete-case analysis and may vary because of missing covariate data. Home SHS exposure was modeled without adjustment for indoor smoking at home because of conceptual overlap with the outcome. Poisson regression with log link and standard errors clustered at the school level. APR: adjusted prevalence ratio. SHS: secondhand smoke. VND: 1 million Vietnamese Dong about US$38.

* p<0.05,

** p<0.01,

*** p<0.001.

## DISCUSSION

### Key findings

In this cross-sectional study of non-current smoking students aged 13 to under 18 years in three selected Vietnamese cities, SHS exposure from conventional cigarettes was common, particularly in public places. Overall, 67.2% of students reported any SHS exposure in the past 7 days, while 22.0% reported home SHS exposure, 50.4% indoor public-place SHS exposure, and 57.5% outdoor public-place SHS exposure. In the fully adjusted model, indoor smoking at home was the strongest factor associated with any SHS exposure, highlighting the central role of the household smoking environment. Setting-specific analyses suggested different exposure patterns across contexts: home SHS exposure was more common in Da Nang and less common in higher grades, whereas indoor and outdoor public-place SHS exposure were more common among older students and those with higher weekly allowance.

### Magnitude and setting-specific pattern of SHS exposure

The overall prevalence of SHS exposure in this study was high and is broadly consistent with international evidence showing that adolescent SHS exposure remains widespread. A pooled analysis of Global Youth Tobacco Survey data from many countries reported that 71.7% of adolescents were exposed to SHS in any place, 35.0% at home, and 46.1% in other enclosed public places^5^. Global trend analyses have similarly shown that, although SHS exposure at home has declined in many settings, exposure in public places has often remained stable or increased, indicating persistent gaps in protection for adolescents^6,10^. Our finding that public-place exposure exceeded home exposure is therefore plausible and suggests that adolescents in large Vietnamese cities continue to encounter smoking frequently in everyday social environments. This setting-specific pattern is also consistent with evidence from other countries showing that distinguishing indoor and outdoor public exposure adds important detail. In a study of US middle- and high-school students, 60.6% reported indoor or outdoor SHS exposure in public places, and more than half reported outdoor SHS exposure, underscoring the continued importance of exposure outside the home^11^. In our study, outdoor public-place SHS exposure was the most common setting-specific exposure, followed by indoor public-place exposure, which supports the view that adolescent exposure is shaped not only by household smoking but also by broader public and social environments.

### Household smoking environment as a key determinant

Our strongest finding was the role of indoor smoking at home. Students reporting indoor smoking at home had a substantially higher prevalence of any SHS exposure, and indoor smoking at home was also strongly associated with both indoor and outdoor public-place SHS exposure. This pattern is consistent with WHO guidance that there is no safe level of SHS exposure and that 100% smoke-free environments are the only effective protection^21^. It also aligns with wider evidence that the home remains a major site of child and adolescent tobacco smoke exposure and that family smoking is a key determinant of SHS burden among children^2,12^. Our findings also suggest that household smoking may function as more than a direct indoor exposure source. Indoor smoking at home may indicate a broader smoking-normalized environment in which adolescents are more likely to encounter SHS beyond the household, including in public settings. This interpretation is plausible given that exposure patterns in our data were not limited to home SHS alone but extended to public-place exposure as well.

### Urban variation in SHS exposure

The city-specific results also draw attention. In the fully adjusted model for any SHS exposure, students in Ho Chi Minh City had a higher prevalence of exposure to SHS than those in Hanoi, whereas the excess in Da Nang was attenuated after adjustment for indoor smoking at home. In the setting-specific models, Da Nang was associated with higher home SHS exposure, while Ho Chi Minh City was associated with higher outdoor public-place SHS exposure. This pattern is compatible with existing Vietnamese evidence showing that SHS levels in public places declined after implementation of smoke-free legislation but remained substantial, especially where enforcement was incomplete^13^. In one Vietnamese assessment conducted five years after the implementation of the tobacco control law, SHS concentrations in monitored public venues were reduced by roughly 45%, but the authors concluded that stronger enforcement was still needed to eliminate SHS in public places^13^. Our results therefore suggest that variation between large Vietnamese cities may reflect different combinations of household smoking practices and public exposure environments rather than a single uniform urban effect.

### Age and social patterning of exposure

Grade level showed a clear setting-specific pattern. In the main model, Grade 12 students had a higher prevalence of any SHS exposure than Grade 8 students. In the secondary analyses, higher grades were associated with greater exposure in indoor and outdoor public places, whereas home SHS exposure was lower in Grades 10–12 than in Grade 8. A similar age-related shift toward public exposure is plausible because older adolescents generally have greater mobility and spend more time in social, leisure, and commercial environments where smoking occurs^6,11^. In contrast, younger students may spend more time in family settings, which could explain the relatively greater importance of home exposure in lower grades.

Weekly allowance was also consistently associated with SHS exposure. Compared with students reporting no allowance, those with modest or moderate weekly allowance had a higher prevalence of any SHS exposure and a higher prevalence of setting-specific exposure, particularly in public places. This likely reflects increased autonomy, mobility, and time spent in exposure-prone environments rather than a direct economic effect alone. The allowance gradient remained after adjusting for indoor smoking at home, suggesting that public and social exposure contexts contribute independently to adolescent SHS exposure. This interpretation is consistent with the broader observation that public-place SHS remains common even where smoke-free laws exist, particularly in leisure and hospitality settings^11,13^.

### Public health implications

The findings should be interpreted as evidence of important setting-specific patterns of SHS exposure among non-current smoking adolescents in major Vietnamese cities, rather than evidence of causal effects. The high occurrence of exposure in both household and public-place contexts suggests that future studies should examine how family smoking practices, adolescent mobility, social environments, and local enforcement conditions jointly shape SHS exposure. Longitudinal studies would be useful to clarify temporal relationships between household smoking environment, adolescent mobility, and public-place exposure. Implementation studies are also needed to assess how smoke-free policies and smoke-free home initiatives operate in settings frequently accessed by adolescents. Such evidence would help strengthen the basis for designing and evaluating strategies to reduce adolescent SHS exposure in Vietnam.

### Strengths and limitations

This study has several strengths. First, it used data from a large school-based survey of non-current smoking adolescents in three major Vietnamese cities, allowing assessment of SHS exposure among students who were not active cigarette smokers. Second, the analysis distinguished exposure at home, in indoor public places, and in outdoor public places, providing a more detailed understanding of where adolescents encounter SHS. Third, the use of adjusted prevalence ratios with school-level clustered robust standard errors allowed associations to be estimated in a way that was appropriate for common binary outcomes and the clustered sampling design.

Several limitations should be considered when interpreting these findings. First, because this was a cross-sectional study, the observed associations cannot be interpreted as causal. Second, SHS exposure and related household smoking variables were measured using self-reported questionnaire data, so recall error, misclassification, and social desirability bias are possible. Because smoking and tobacco-related exposure may be socially sensitive among students, exposure or household smoking behaviors may have been underreported. Selection bias is also possible because the analysis included only school-attending adolescents who completed valid questionnaires; adolescents absent from school or outside the school system may have different exposure patterns. Third, the study population included only school-attending adolescents in three selected large cities; therefore, the findings should be interpreted as most relevant to urban students in major Vietnamese cities, rather than to all adolescents in Vietnam. In particular, these results may not be generalizable to adolescents who are out of school, to those living in smaller cities or rural areas, or to provinces with different tobacco-use patterns, enforcement conditions, and social environments. Fourth, the analytic dataset did not include sampling weights, so prevalence estimates should be interpreted as sample-based estimates rather than population-representative estimates for the three cities. Finally, residual confounding may still have affected the observed associations. Although the models adjusted for key sociodemographic factors and indoor smoking at home, the analysis could not account for several potentially relevant factors, including parental smoking status, number of smokers in the household, peer smoking, school-level smoking norms, neighborhood tobacco retail density, and enforcement of smoke-free regulations in specific venues. These unmeasured factors may partly explain the observed associations, particularly those related to public-place exposure, grade level, city, and weekly allowance.

## CONCLUSIONS

SHS exposure from conventional cigarettes was common among non-current smoking students in three selected cities in Vietnam. Exposure was strongly associated with indoor smoking at home, and differences were observed by city, grade, and weekly allowance. The setting-specific analyses suggest that home exposure remains important, while public-place exposure is highly prevalent and may become more prominent as adolescents grow older. These findings highlight the importance of household and public-place contexts for adolescent SHS exposure, but further longitudinal and implementation studies are needed to clarify causal pathways and strengthen the evidence base for strategies to reduce SHS exposure in home and public environments. The findings also remain relevant to ongoing efforts to implement WHO FCTC Article 8 protections.

## Data Availability

The data supporting this research are available from the authors on reasonable request.
